# Are all measures of liver $Kp_{uu}$ a function of $F_{H}$, as determined following oral dosing, or have we made a critical error in defining hepatic drug clearance?

**DOI:** 10.1016/j.ejps.2024.106753

**Published:** 2024-03-22

**Authors:** L.Z. Benet, J.K. Sodhi

**Affiliations:** Department of Bioengineering and Therapeutic Sciences, Schools of Pharmacy and Medicine, University of California San Francisco, San Francisco, CA, USA

**Keywords:** Kp_uu_, Hepatic bioavailability, Liver to blood ratio, Protein binding

## Abstract

Here we present, utilizing universally accepted relationships for hepatic clearance at steady state, that for all models of hepatic elimination the ratio of unbound liver drug concentration to unbound systemic blood concentration, Kp_uu_, is a function of or related to the hepatic bioavailability for that drug, F_H_. According to the derivation for the well-stirred model, Kp_uu_ can never exceed unity, can frequently be a function of hepatic blood flow, and is equivalent to the value of F_H_ as determined following oral dosing. For the parallel tube model, Kp_uu_ will not equal F_H_ but will be a function of F_H_ and will also never be a value greater than 1. When hepatic clearance is rate limited by basolateral transporters, Kp_uu_ will be less than 1, and less than F_H_. We believe that such outcomes are highly unlikely, and that the error arises from a basic assumption concerning hepatic clearance that leads to the mechanistic models of hepatic elimination, the well-stirred, parallel tube and dispersion models. That basic assumption is that the steady-state systemic concentration multiplied by the hepatic systemic clearance is equal to the product of the average unbound liver steady-state concentration and the intrinsic hepatic clearance (C_ss_ · CL = C_H,u_ · CL_int_). Calculations of Kp_uu_ and F_H_ based on present methods of analysis provide a strong argument as to why this universally accepted relationship is not correct. Alternatively, we have shown in recent publications that hepatic clearance may be adequately determined based on Kirchhoff’s Laws where no assumption of the above equality concerning hepatic intrinsic clearance is required, and where Kp_uu_ is independent of hepatic extraction ratio and F_H_.

## 1. Introduction

In 2018, our laboratory (Benet et al., 2018b) began questioning the relevance and accuracy of the mechanistic models of hepatic elimination, i.e., the well-stirred model (WSM), the parallel tube model (PTM) and the dispersion models (DM). Our position was vigorously countered (Rowland and Pang, 2018; Pang et al., 2019). We continued to examine the issue based on experimental data generated in our laboratory (Wang and Benet, 2019) and from numerous other laboratories (Sodhi et al., 2020a), as well as from theoretical perspectives (Benet and Sodhi, 2020; Benet et al., 2021, 2022; Benet and Sodhi, 2023). These presentations were also vigorously countered (Jusko and Li, 2022; Rowland and Pang, 2022; Rowland et al. 2022; Rowland et al., 2023). At the September 2022 ISSX/MDO meeting, a debate was organized between Professors Benet and Pang under the title “Perspectives on Long-Held Clearance Concepts”, however, this still did not settle the question in the minds of many. Here we present a previously unrecognized relationship between liver $Kp_{uu}$, the ratio of unbound liver drug concentration to unbound systemic blood concentration, and $F_{H}$, the model independent measure of the fraction of an oral dose that escapes elimination on a drug’s first pass through the liver, for the mechanistic models of hepatic elimination. Given that such a relationship between liver $Kp_{uu}$ and $F_{H}$ and the limitations it imposes are not realistic, we present here a compelling argument for the invalidity of using mechanistic models of hepatic elimination when only systemic drug concentrations are measured. The $Kp_{uu}$ concept is familiar to many scientists in terms of its relevance with respect to the brain (Loryan et al., 2022), a non-eliminating organ, and therefore not considered in the present evaluation.

Beginning in the 1970s, hepatic clearance equations were developed based on a specific mechanistic model, initially the WSM (Rowland et al., 1973; Wilkinson and Shand, 1975). However, the conditions for the WSM have been believed to be non-physiological. Yet, when seemingly more physiologically sound models were proposed, initially the PTM (Pang and Rowland, 1977) and DM (Roberts and Rowland, 1986), the WSM-derived equation continued to best represent the experimental data (Pang et al., 2019; Sodhi et al., 2020a). We reasoned that the equations following the WSM might be valid for reasons other than those assumed in the WSM derivation. We then reported that we could simply derive the equation believed to be the WSM using Kirchhoff’s Laws (Patcher et al., 2022; Benet and Sodhi, 2023) independent of any mechanistic model of hepatic elimination, where for rate defining processes in series, i.e., hepatic blood flow ($Q_{H}$) and the intrinsic clearance of unbound drug ($CL_{int}$) multiplied by the fraction of drug unbound in blood ($f_{uB}$), the addition of the inverse of each rate defining process would be equal to the inverse of hepatic clearance ($CL_{H}$) .


(1) $$\frac{1}{CL_{H}}=\frac{1}{Q_{H}}+\frac{1}{f_{uB}\cdot CL_{int}}$$

Solving Eq. (1) gives

(2) $$CL_{H}=Q_{H}\cdot \frac{f_{uB}\cdot CL_{int}}{Q_{H}+f_{uB}\cdot CL_{int}}$$

Very simply, and without differential equations, we have shown that it is possible to derive the coefficient of proportionality (clearance or rate constants) between the rate of reaction and the driving force for that reaction, concentrations or amounts. The coefficients of proportionality (clearance for concentration driven reactions and rate constants for amount driven reactions) are added for parallel processes, and for in series processes the inverse of the of the individual in series coefficients of proportionality equals the inverse of the overall clearance or rate constant. It is critical to recognize that the inclusion of the coefficients of proportionality within the Kirchhoff’s Laws derivations are valid only for rate defining processes, that is, processes that under certain conditions can be singly equal to the total clearance parameter. For example, in hepatic elimination: hepatic blood flow, metabolic intrinsic clearance or basolateral transport; in kidney elimination: kidney blood flow, glomerular filtration or renal tubular transport. We argued that Eq. (2) was not the WSM, but rather the general equation describing clearance when only systemic concentrations are measured and basolateral transporters are not considered (Patcher et al., 2022; Benet and Sodhi, 2023). We further proposed that mechanistic models of hepatic elimination provide no valid representation of hepatic clearance, since those values are based on systemic concentration measurements. Although we have introduced the Kirchhoff’s Laws approach, this manuscript only details the unusual outcomes relating to $Kp_{uu}$ and $F_{H}$ when considering the present mechanistic models of hepatic elimination.

## 2. Methods and theoretical analysis

The primary mass balance equation that serves today and for the past 50 years as the basis for the derivation of the hepatic clearance-intrinsic clearance relationship for the WSM, PTM and DM, as well as for the characterization of hepatic elimination in physiologic based pharmacokinetic (PBPK) models at steady-state is

(3) $$CL_{Blood}\cdot C_{Blood}=Q_{H}\cdot (C_{in}-C_{out})=CL_{int}\cdot C_{H,u}$$

where $C_{Blood}$ is the steady-state concentration of total drug in the blood, $CL_{Blood}$ is blood clearance, $C_{in}$ and $C_{out}$ are the blood concentrations of total drug (unbound plus bound) entering and leaving the liver, respectively, and $C_{H,u}$ is the average concentration of unbound drug within the liver, as recently reviewed by Li and Jusko (2022). Eq. (3) may also be written in terms of unbound steady-state blood concentration ($C_{Blood,u}$) and unbound hepatic blood clearance ($CL_{Blood,u}$).

(3a) $$CL_{Blood,u}\cdot C_{Blood,u}=Q_{H}\cdot (C_{in}-C_{out})=CL_{int}\cdot C_{H,u}$$

When mechanistic models of hepatic elimination are considered, Eq. (3a) can be rewritten as proposed by Pang and Rowland (1977) and Roberts and Rowland (1986) for all mechanistic models of hepatic elimination as

(3b) $$CL_{Blood,u}\cdot C_{Blood,u}=CL_{int,WSM}\cdot C_{H,u}=CL_{int,PTM}\cdot C_{H,u}=CL_{int,DM}\cdot C_{H,u}$$

with each mechanistic model assuming a differing $C_{H,u}$.

### 2.1. Deriving $Kp_{uu}$ for the WSM equation of hepatic clearance when basolateral transporters are not considered

It is possible to derive the in vivo steady-state relationship between $Kp_{uu}$ (i.e., $\frac{C_{H,u}}{C_{Blood,u}}$) and the clearance-related parameters for each model by substituting the unbound blood clearance into Eq. (3b). For the WSM derivation (equivalent to Eq. (2))

(4) $$Kp_{uu,WSM}=\frac{C_{H,u}}{C_{Blood,u}}=\frac{CL_{Blood,u,WSM}}{CL_{int}}=\frac{\frac{Q_{H}\cdot CL_{int}}{Q_{H}+f_{uB}\cdot CL_{int}}}{CL_{int}}=\frac{Q_{H}}{Q_{H}+f_{uB}\cdot CL_{int}}$$

Thus, based on Eq. (3), for the WSM hepatic clearance, in vivo $Kp_{uu}$ can never be greater than unity, and except for very low clearance drugs $Kp_{uu}$ is a function of hepatic blood flow.

But there is a further outcome. Independent of the model of hepatic elimination, the fraction of an oral dose that escapes first pass hepatic elimination, $F_{H}$, is calculated by Eq. (5),

(5) $$F_{H}=1-ER=1-\frac{CL_{Blood}}{Q_{H}}$$

where ER is the hepatic extraction ratio. For the WSM, $F_{H}$ can be calculated as

(5a) $$F_{H}=1-\frac{CL_{Blood,WSM}}{Q_{H}}=1-\frac{\frac{Q_{H}\cdot f_{uB}\cdot CL_{int}}{Q_{H}+f_{uB}\cdot CL_{int}}}{Q_{H}}=\cdot \frac{Q_{H}}{Q_{H}+f_{uB}\cdot CL_{int}}$$

for a drug only eliminated by hepatic processes. Thus, based on Eq. (3), for the WSM hepatic clearance derivation, $Kp_{uu}=F_{H}$. Although not previously reported, the outcome is consistent with the WSM, where $C_{H,u}$ is assumed to equal $C_{out,u}$. Thus, for the WSM both $Kp_{uu}$ and $F_{H}$ equal $\frac{C_{out}}{C_{in}}$. It is worth noting that the determination of these two equivalent parameters results from completely different measurements. $Kp_{uu}$ requires measurements of liver and systemic fluid concentrations at steady-state and measurements of fraction unbound to protein in each tissue via several methodologies as we reviewed (Sodhi et al., 2020b). In contrast, even though we have shown the relationship in terms of intrinsic clearance in Eq. (5a), $F_{H}$ is experimentally determined as a measure of hepatic clearance, i.e., the available single dose divided by the systemic exposure (AUC_0→∞_) minus any renal clearance.

### 2.2. Deriving $Kp_{uu}$ for the parallel tube model equation of hepatic clearance

Recognizing that the steady-state average concentration within the liver for the PTM is $\frac{C_{in}-C_{out}}{ln\frac{C_{in}}{C_{out}}}$ (Jusko and Li, 2022) and $ER=\frac{C_{in}-C_{out}}{C_{in}}=1-F_{H}$, independent of mechanistic hepatic elimination models, the equation for $Kp_{uu,PTM}$ can be written directly

(6) $$Kp_{uu,PTM}=\frac{C_{H,u}}{C_{Blood,u}}=\frac{f_{uB}\cdot \frac{C_{in}-C_{out}}{ln\frac{C_{in}}{C_{out}}}}{f_{uB}\cdot C_{in}}=\frac{\frac{C_{in}-C_{out}}{C_{in}}}{ln\frac{C_{in}}{C_{out}}}=\frac{1-F_{H}}{ln\frac{1}{F_{H}}}$$

**Fig. 1.**
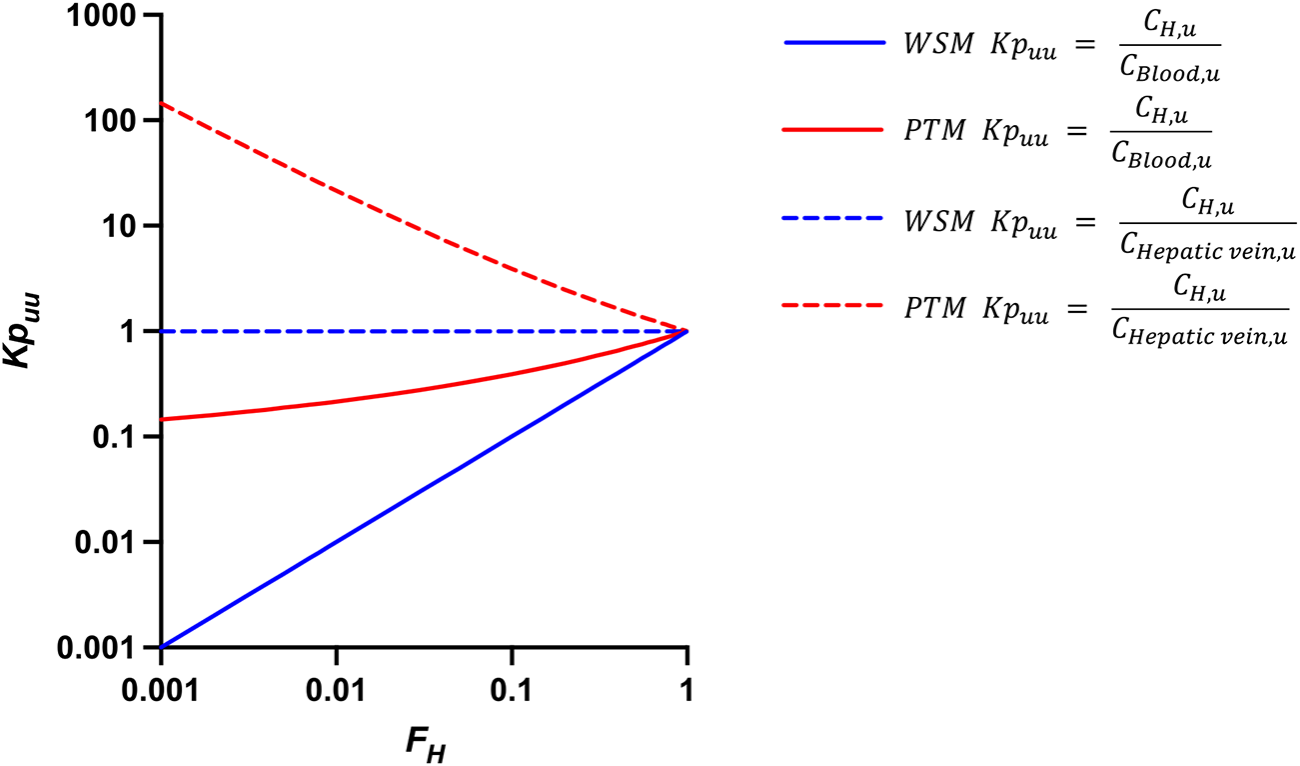
Theoretical relationship between $Kp_{uu}$ and $F_{H}$ for the WSM and PTM if one accepts Eq. (3) as valid. Solid lines when $Kp_{uu}$ is defined as the steady-state ratio of average unbound drug concentration in the liver to the unbound systemic concentration. Dashed lines when $Kp_{uu}$ is defined as the steady-state ratio of average unbound drug concentration in the liver to the unbound hepatic vein concentration. The WSM is depicted in blue and the PTM in red. If similar calculations were made for the DM, the resulting lines would be intermediate to those for the WSM and PTM dependent on the dispersion number.

For the PTM, $Kp_{uu,PTM}\neq F_{H}$, but it is a function of $F_{H}$. When a drug exhibits a very low extraction ratio, $Kp_{uu,PTM}$ is just less than unity (e.g., $F_{H}=0.99$ then $Kp_{uu,PTM}=0.995$). When the extraction ratio is very high, $Kp_{uu,PTM}$ must be less than one to a greater degree (e.g., $F_{H}=0.01$ then $Kp_{uu,PTM}=0.215$). If one accepts Eq. (3) as valid, then the relationship between $Kp_{uu}$ and $F_{H}$ for the WSM and PTM are depicted by the solid lines in Fig. 1, and $Kp_{uu}$ for both models are always ≤ 1.0. Above, we indicated that the finding of $Kp_{uu,WSM}=F_{H}$ may be justified for the WSM, but there appears to be no justification for the Eq. (6) relationship.

### 2.3. The definition of $Kp_{uu}$

Above, we have defined $Kp_{uu}$ in the usual manner, as the in vivo steady-state ratio of the unbound concentration within the liver to the unbound systemic blood concentration (e.g., Di et al., 2021). Recently Li and Jusko (2022) have proposed that $Kp_{ss,Li\;\&\;jusko}$, the ratio of total drug concentrations, should rather be the ratio of total drug concentration in the liver to total drug concentration in the blood exiting the liver, $C_{Hepatic\;vein}=C_{out}$. Although they did not address this directly, we assume that they would then calculate $Kp_{uu,ss}$, in the same manner (i.e., $Kp_{uu,ss,Li\;\&\;Jusko}=\frac{C_{H,u}}{C_{out,u}}$)

For such an assumption the $Kp_{uu,ss,Li\;\&\;Jusko}$ values for the WSM with and without transporters would equal 1.0 for all drugs, since the WSM assumes $C_{H,u}=C_{out,u}$. It is difficult to see how such an outcome based on the Li & Jusko assumption provides any value, in that $Kp_{uu,ss,Li\;\&\;Jusko}$ is the same for all drugs when systemic concentrations are described by the WSM.

Since $C_{out}=F_{H}\cdot C_{in}$, the $Kp_{uu}$ relationship for the PTM based on the Li and Jusko assumption, independent of whether basolateral transporters are included or not, would be:

(7) $$Kp_{uu,PTM,Li\;\&\;Jusko}=\frac{C_{H,u}}{C_{Hepatic\;vein,u}}=\frac{f_{uB}\cdot \frac{C_{in}-C_{out}}{ln\frac{C_{in}}{C_{out}}}}{f_{uB}\cdot C_{out}}=\frac{\frac{C_{in}-C_{out}}{C_{in}}\cdot \frac{C_{in}}{C_{out}}}{ln\frac{C_{in}}{C_{out}}}=\frac{\frac{1-F_{H}}{F_{H}}}{ln\frac{1}{F_{H}}}$$

For the Li and Jusko (2022) assumption, $Kp_{uu,PTM,Li\&Jusko}$ is always ≥ 1.0. For a low extraction ratio drug, there is essentially little change in $Kp_{uu,PTM,Li\&Jusko}$ between Eqs. (6) and (7), but now it is slightly greater than unity (e.g., $F_{H}=0.99$ then $Kp_{uu,PTM,Li\;\&\;Jusko}=1.005$), and for a high extraction ratio drug $Kp_{uu,PTM,Li\;\&\;Jusko}$ now becomes much greater than 1.0 (e.g., $F_{H}=0.01$ then $Kp_{uu,PTM,Li\;\&\;Jusko}=21.5$). If one accepts Eq. (3) as valid, and the proposal of Li and Jusko (2022) that partition coefficients should be liver steady-state concentration to hepatic vein steady-state concentration, then the relationship between $Kp_{uu}$ and $F_{H}$ for the WSM and PTM are depicted by the dashed lines in Fig. 1.

### 2.4. Deriving $Kp_{uu}$ for the extended clearance model of hepatic clearance when basolateral transport is the rate limiting step in hepatic elimination

We approach this topic based on the Extended Clearance Model (ECM) equation derivation presented in many papers as we reviewed (Benet et al., 2018a):

(8) $$CL_{Blood,ECM}=\frac{Q_{H}\cdot PS_{influx}\cdot f_{uB}\cdot CL_{int}}{Q_{H}\cdot \left(CL_{int}+PS_{efflux}\right)+PS_{influx}\cdot f_{uB}\cdot CL_{int}}$$

where $PS_{influx}$ and $PS_{efflux}$ are the total hepatic (active plus passive) intrinsic basolateral influx and efflux clearances, respectively. Substituting Eq. (8) into Eq. (3) gives

$$C_{Blood}\cdot \frac{Q_{H}\cdot PS_{influx}\cdot f_{uB}\cdot CL_{int}}{Q_{H}\cdot \left(CL_{int}+PS_{efflux}\right)+PS_{influx}\cdot f_{uB}\cdot CL_{int}}=C_{H,u}\cdot CL_{int}$$

which can be rearranged to:

(9) $$\frac{C_{H,u}}{C_{Blood,u}}=Kp_{uu,ECM}=\frac{Q_{H}\cdot PS_{influx}}{Q_{H}\cdot \left(CL_{int}+PS_{efflux}\right)+PS_{influx}\cdot f_{uB}\cdot CL_{int}}\;\;=\frac{PS_{influx}}{\left(CL_{int}+PS_{efflux}\right)+\frac{PS_{influx}\cdot f_{uB}\cdot CL_{int}}{Q_{H}}}$$

Frequently it is proposed that when $Q_{H}\gg PS_{influx}\cdot f_{uB}\cdot CL_{int}$, the more usual form for the $Kp_{uu,ECM}$ equation is seen (e.g., Di et al., 2021)

(10) $$Kp_{uu,ECM}=\frac{PS_{influx}}{CL_{int}+PS_{efflux}}$$

Thus, it would appear that $Kp_{uu,ECM}$ can range from a very high number to a very low number depending on the relative values of the numerator and denominator of Eq. (10). And this is true.

However, if hepatic uptake is the rate limiting step for elimination, which is proposed for the great majority of ECCS (Extended Clearance Classification) class 1B and 3B acidic drugs (Varma et al., 2015) such as HMG-CoA reductase inhibitors (i.e., statins), then $PS_{influx}$ must be much less than $\left(CL_{int}+PS_{efflux}\right)$ and $Kp_{uu,ECM}$ must always be much less than 1 for these drugs.

Substituting Eq. (8) into Eq. (5) gives

(11) $$\;F_{H}=1-ER=1-\frac{CL_{Blood}}{Q_{H}}=1-\frac{\frac{Q_{H}\cdot PS_{influx}\cdot f_{uB}\cdot CL_{int}}{Q_{H}\cdot \left(CL_{int}+PS_{efflux}\right)+PS_{influx}\cdot f_{uB}\cdot CL_{int}}}{Q_{H}}\;=\frac{Q_{H}\cdot \left(CL_{int}+PS_{efflux}\right)}{Q_{H}\cdot \left(CL_{int}+PS_{efflux}\right)+PS_{influx}\cdot f_{uB}\cdot CL_{int}}=\frac{\left(CL_{int}+PS_{efflux}\right)}{\left(CL_{int}+PS_{efflux}\right)+\frac{PS_{influx}\cdot f_{uB}\cdot CL_{int}}{Q_{H}}}$$

Now by comparing Eq. (11) to Eq. (9), we see that for the ECM equation when hepatic basolateral transport is rate limiting, not only is $Kp_{uu,ECM}$ always less than 1.0, but $Kp_{uu}$ is always less than $F_{H}$.

Thus, in all cases detailed above for the WSM and PTM, whether a) hepatic basolateral transport is not relevant or b) when basolateral hepatic uptake is the rate limiting step for hepatic clearance, then $Kp_{uu}\leq F_{H}$, based on Eq. (3) for the usual definition of in vivo $Kp_{uu}$(Di et al., 2021). As a result, the value of $Kp_{uu}$ can never exceed unity.

As we note, detail, and ask in the Discussion, what knowledgeable drug metabolism/pharmacokinetics scientist would believe any of these outcomes showing that there is a relationship between $Kp_{uu}$, $F_{H}$ and ER, now that they have been explicitly presented and depicted here for the first time?

Until now, no recognized measures have been available to differentiate our position that the mechanistic models of hepatic elimination are not useful in defining clearance relationships for drugs when only systemic concentrations are measured (Benet et al., 2018b; Benet and Sodhi, 2020, 2023; Benet et al., 2021; ; Patcher et al., 2022). Our field’s present analysis accepts Eq. (3), which leads directly to what we consider unbelievable relationships between $Kp_{uu}$ and measures of $F_{H}$. For the WSM, without including transporter activity, Eq. (3) leads to $Kp_{uu}=F_{H}$ with an outcome that $Kp_{uu}$ can never exceed unity. An equally preposterous outcome is found using the potential Li and Jusko (2022) definition of $Kp_{uu}$, which relates unbound hepatic concentrations to unbound concentration in the hepatic vein. Under that condition for the WSM relation, $Kp_{uu}=1.0$ for all drugs, as the WSM assumes $C_{H,u}=C_{out,u}$. Accepting the validity of Eq. (3), we also derived the relationship between $Kp_{uu}$ and $F_{H}$ for the PTM with outcomes that we believe no knowledgeable scientist will accept. That is, $Kp_{uu}$ is a function of whether the drug is a low or high ER compound, independent of any structural molecule characteristics. All of these unbelievable relationships are depicted in Fig. 1, and we suggest that knowledgeable pharmaceutical scientists will also concur that such relationships are unlikely to be valid. We have not analyzed the much more complicated DM; the results would be numerically different than the PTM analysis depending on the dispersion number chosen, but the outcome will be the same; $Kp_{uu}$ will be a function of $F_{H}$ with lines intermediate to those in Fig. 1 for the WSM and PTM.

If the results from the analysis here are considered, there must be an error in Eq. (3). We approach this error from two different perspectives. First, we have expanded Eq. (3a) to include the clearance of unbound drug in the liver at steady-state based on liver concentrations ($CL_{Liver,u}$) multiplied by the steady-state hepatic unbound concentration ($C_{H,u}$)

(12) $$CL_{Blood,u}\cdot C_{Blood,u}=Q_{H}\cdot \left(C_{in}-C_{out}\right)=CL_{Liver,u}\cdot C_{H,u}\neq CL_{int}\cdot C_{H,u}$$

The third product in Eq. (12) ($CL_{Liver,u}\cdot C_{H,u}$), is added here, as we proposed in 2018 (Benet et al., 2018b). When a drug is only eliminated by metabolism, at steady-state the rate of elimination of drug in the systemic circulation must equal the rate of elimination of drug in the liver. Note the unbound hepatic clearance determined based on systemic concentrations (dividing each side of Eq. (2) by $f_{uB}$) will only equal $CL_{Liver,u}$, the hepatic clearance based on liver concentrations when $Kp_{uu}=1$. By examining the third and fourth products in Eq. (12), the error in defining hepatic disposition models made for the last 50 years becomes obvious. It has been assumed that liver clearance is only a function of the metabolic intrinsic clearance, and that for each hepatic disposition model, the average unbound hepatic concentration can be calculated by considering only this value. But why should that be true? If blood clearance is rate limited or affected by hepatic blood flow, shouldn’t liver clearance also be rate limited or affected by hepatic blood flow?

Alternatively, since Eq. (3a) defines elimination in the liver, average $C_{H,u}$ must always be less than $C_{Blood,u}$. Therefore, $f_{uB}\cdot CL_{int}$ must always be greater than $CL_{Blood,u}$. One can easily confirm this. For $Q_{H}=1500\;\text{ml}∕\text{min}$ and any non-zero value of $f_{uB}\cdot CL_{int}$ in either what was considered the WSM (Eq. (2)) or for the PTM (Eq. (13)), $f_{uB}\cdot CL_{int}>CL_{Blood}$.


(13) $$CL_{Blood,PTM}=Q_{H}\cdot \left(1-e^{-\frac{f_{uB}\cdot CL_{int}}{Q_{H}}}\right)$$

Since $CL_{Blood}\leq CL_{Blood,u}$, if the Eq. (3) assumption is correct then $Kp_{uu}$ must be less than 1 (or equal to 1) for all drugs. Does our field really believe this?

The validity of Eq. (3) is often justified based on mass balance (Rowland and Pang, 2018 and 2022; Rowland et al., 2022 and 2023). However, mass balance is a necessary but not a sufficient validity criterion by itself. For example, Eq. (14) also maintains mass balance, just like Eq. (3).


(14) $$CL_{Blood,u}\cdot C_{Blood,u}=Q_{H}\cdot \left(C_{in}-C_{out}\right)=Q_{H}\cdot C_{H,u}$$

Neither Eq. (14) nor Eq. (3) are valid for almost all drugs, but when systemic clearance is rate limited by hepatic blood flow the relationship cannot be differentiated from Eq. (14), and when systemic clearance is very much smaller than hepatic blood flow the relationship cannot be differentiated from Eq. (3). As we have demonstrated (Patcher et al., 2022; Benet and Sodhi, 2023), the Kirchhoff’s Laws derivation of hepatic clearance makes no assumption concerning the mechanistic basis of liver elimination and it is not valid to define the clearance rate of drug as measured in the blood in terms of any intrahepatic relationship. There is no general validity to Eq. (3) and therefore the derivations of the WSM, PTM and DM also have no relevance when only systemic concentrations are measured. And thus, there is, in fact, no valid relationship between $Kp_{uu}$ and $F_{H}$, which had only resulted here by accepting Eq. (3) as valid.

### 3. Conclusions

The present manuscript demonstrates that the basic assumption that the product of the steady-state systemic blood concentration multiplied by the systemic blood clearance is equal to the product of the unbound drug concentration in the liver multiplied by the liver intrinsic clearance (i.e., $C_{Blood}\cdot CL_{Blood}=C_{H,u}\cdot CL_{int}$) leads directly to the result that $Kp_{uu}$ is a function of $F_{H}$. There is no reason to believe that such an outcome is true, and since this equality serves as the basis for the WSM, PTM and DM, there is no rationale for defining drug clearance in terms of these hepatic mechanistic models when only systemic concentrations are measured. Hepatic bioavailability, $F_{H}$, is adequately and correctly calculated by Eq. (5) independent of any mechanistic model of hepatic elimination. The model independent relationships between intrinsic clearance, hepatic blood flow and hepatic basolateral transporters can be adequately defined using Kirchhoff’s Laws for rate defining processes in series (Patcher at al., 2022; Benet and Sodhi, 2023). There should be no expectation that a measure of liver to blood partition of unchanged drug at steady-state is in any way related to a measure of hepatic bioavailability, which only results, as shown here by assuming that hepatic clearance is not influenced by hepatic blood flow, which is inherent in Eq. (3), (3a) and (3b) Finally, the proposal of Li and Jusko (2022) that partition should be evaluated based on liver to exiting blood concentration ratios provides no useful outcomes when one is determining $Kp_{uu}$.

## Data Availability

No data was used for the research described in the article.

## Abbreviations:

C_Blood_: steady-state concentration of total drug in blood
C_Blood,u_: steady-state concentration of unbound drug in blood
C_H_: steady-state concentration of total drug in the liver
C_H,u_: steady-state concentration of unbound drug in the liver
C_in_: blood concentration entering the liver
C_out_ and C_Hepatic vein_: blood concentration exiting the liver
CL_Blood_: blood clearance
CL_Blood,u_: unbound blood clearance
CL_H,u_: hepatic clearance determined based on systemic unbound concentrations
CL_int_: intrinsic hepatic clearance
CL_Liver,u_: hepatic clearance based on unbound liver concentrations
DM: dispersion model
ECCS: Extended Clearance Classification System
ECM: Extended Clearance Model
ER: hepatic extraction ratio
F_H_: hepatic bioavailability
f_uB_: fraction of drug unbound in the blood
KL: Kirchhoff’s Laws
Kp_ss_: Li & Jusko ratio at steady-state of total liver drug concentration to total drug concentration exiting the liver
Kp_ss,uu_: Li & Jusko, ratio at steady-state of unbound liver drug concentration to unbound drug concentration exiting the liver
Kp_uu_: in vivo ratio at steady-state of unbound liver drug concentration to unbound systemic blood concentration
PS_efflux_: intrinsic basolateral efflux clearance
PS_influx_: intrinsic basolateral influx clearance
PTM: parallel tube model
Q_H_: hepatic blood flow
WSM: well-stirred model
